# An electronic warning system helps reduce the time to diagnosis of sepsis

**DOI:** 10.5935/0103-507X.20180059

**Published:** 2018

**Authors:** Glauco Adrieno Westphal, Aline Braz Pereira, Silvia Maria Fachin, Geonice Sperotto, Maurício Gonçalves, Lucimeri Albino, Rodolfo Bittencourt, Vanessa de Rossi Franzini, Álvaro Koenig

**Affiliations:** 1 Centro Hospitalar Unimed - Joinville (SC), Brasil.

**Keywords:** Sepsis/diagnosis, Hospital mortality, Medical order entry systems, Alert

## Abstract

**Objective:**

To describe the improvements of an early warning system for the identification of septic patients on the time to diagnosis, antibiotic delivery, and mortality.

**Methods:**

This was an observational cohort study that describes the successive improvements made over a period of 10 years using an early warning system to detect sepsis, including systematic active manual surveillance, electronic alerts via a telephonist, and alerts sent directly to the mobile devices of nurses. For all periods, after an alert was triggered, early treatment was instituted according to the institutional sepsis guidelines.

**Results:**

In total, 637 patients with sepsis were detected over the study period. The median triage-to-diagnosis time was reduced from 19:20 (9:10 - 38:15) hours to 12:40 (2:50 - 23:45) hours when the manual surveillance method was used (p = 0.14), to 2:10 (1:25 - 2:20) hours when the alert was sent automatically to the hospital telephone service (p = 0.014), and to 1:00 (0:30 - 1:10) hour when the alert was sent directly to the nurse's mobile phone (p = 0.016). The diagnosis-to-antibiotic time was reduced to 1:00 (0:55 - 1:30) hours when the alert was sent to the telephonist and to 0:45 (0:30 - 1:00) minutes when the alert was sent directly to the nurse's mobile phone (p = 0.02), with the maintenance of similar values over the following years. There was no difference in the time of treatment between survivors and non-survivors.

**Conclusion:**

Electronic systems help reduce the triage-to-diagnosis time and diagnosis-to-antibiotic time in patients with sepsis.

## INTRODUCTION

Sepsis is a common clinical condition associated with a high mortality rate among hospitalized patients and constitutes a leading cause of death worldwide.^(1-4)^ In developing countries, the rate of sepsis-related mortality is almost twice that of developed countries.^(1,5,6)^

Over the last 15 years, the importance of the early introduction of appropriate antibiotics and early control of hemodynamic instability has been demonstrated. However, the authentic precocity of these interventions depends on the capacity to identify patients at risk of sepsis,^(4,7-19)^ and delayed diagnosis is a major obstacle to initiating treatment and reducing mortality.^(14,20)^

Three prospective two-phase cohort studies observed that, although compliance with appropriate antibiotic therapy remained the same over time, mortality fell significantly after implementation of active surveillance that reduces the time to identify patients with suspected sepsis.^(17,18)^ The implementation of institutional strategies based on alert systems to identify sepsis at earlier stages can significantly reduce the time to recognize patients with suspected sepsis and sepsis-related mortality.^(17-19,21,22)^

Clinical signs of systemic inflammatory response syndrome (SIRS) and quick Sepsis-related Organ Failure Assessment (qSOFA) have been proposed as tools for the early detection of sepsis. However, SIRS signs have been criticized for their high sensitivity^(23)^ and, although qSOFA identifies septic patients at high risk of death,^(24,25)^ it lacks sensitivity for the truly early detection of patients at risk of sepsis.^(26-29)^ Three studies assessed the combined use of clinical signs of SIRS (respiratory rate, heart rate, and temperature) and signs of organ dysfunction (SOD) contemplated by the qSOFA (neurological deficit, hypotension, oxygen use, or tachypnea) as initial signs of sepsis. Their results showed a decrease in the time to sepsis diagnosis and its association with a reduction in hospital mortality.^(17,18,30)^

Based on SIRS clinical signs and SOD, some early warning systems have been proposed to detect at-risk patients, such as the Modified Early Warning Score (MEWS)^(31)^ and National Early Warning Score (NEWS).^(32)^ These scores can assist the health care team in identifying patients with higher likelihoods of clinical deterioration^(32)^ and mobilize the health care team at earlier stages of sepsis.^(20)^ Based on electronic medical records containing information on vital signs and SOD, an electronic algorithm can quickly total the final scores of such screening systems. Some authors have reported experiences with electronic alerts that permitted an earlier diagnosis of sepsis and, consequently, earlier and more appropriate treatment.^(33-37)^

Our main purpose was to describe successive improvements in an early warning system used to identify septic patients and to evaluate its effect on the time to sepsis diagnosis, antibiotic delivery, and mortality.

## METHODS

A descriptive, observational cohort study was carried out in a single private hospital with 164 beds located in southern Brazil. Medical health records of septic patients identified between 2005 and 2015 were analyzed. Data from 2008 and 2009 are missing. Septic patients identified in the emergency room and in the wards were included in the analysis. The health care team was encouraged to respond quickly to early warning systems.

### Definitions

Signs of SIRS are clinically detectable signs of SIRS (temperature > 38.5ºC or < 36ºC, heart rate > 90 beats per minute, and respiratory rate > 20 breaths per minute) as well as headache with neck stiffness.^(38,39)^

Signs of organ dysfunctions consisted of the inclusion of clinically detectable SOD, such as systolic blood pressure < 90mmHg or mean arterial pressure (MAP) < 65mmHg, acute encephalopathy (drowsiness, disorientation, confusion, or coma), oliguria, or the need for oxygen supplementation.

Sepsis (formerly called severe sepsis). Clinical signs of infection and at least one sign of organ dysfunction.^(4,26)^

Septic shock corresponded to using the vasopressor requirement to maintain MAP ≥ 65mmHg.^(26)^

Modified early warning score consists of an early warning system used to detect at-risk patients by assessing clinically detectable signs of SIRS and SOD, assigning scores according to the degree of deviation from normal ranges for each of these parameters.^(40)^

6-hour bundle Was the early treatment was instituted according to the institutional sepsis guidelines as follows: determination of serum lactate levels; collection of at least 2 blood samples from different sites for culture; initiation of appropriate antibiotic therapy within 1 hour after diagnosis; in the event of hypotension or serum lactate ≥ 4mmol/L, administration of 30mL/kg of crystalloids; administration of a vasopressor if MAP < 65mmHg after crystalloid infusion.^(14)^ Maintenance of a central venous pressure between 8 and 12mmHg and achievement of central venous oxygen saturation > 70% have not been used since 2011.

### Changes in screening protocols over the years

Our institutional sepsis program began in mid-2005 with the beginning of the Surviving Sepsis Campign (SSC) in Brazil. The first two periods of this program (baseline and active surveillance for clinical signs of infection) were based on manual screening strategies that were reported in a previous publication.^(18)^ Since then, other improvements based on electronic alerts have been made. The following screening methods were used:

- Baseline: from August 2005 to October 2006, all consecutive inpatients with a diagnosis of sepsis were included using the clinical signs of infection suggested in the first two versions of the SSC definitions.^(18,38,39)^
- Manual surveillance for clinical signs of infection: from November 2006 to November 2007, active surveillance of all hospitalized patients was performed. Nursing technicians were trained to identify and report any abnormality of 2 or more signs of SIRS and/or SOD to the ward nurse filling out a specific form. Once 2 or more of these signs were present, the ward nurse requested a medical evaluation.^(18)^
- Indirect electronic alert to telephonist: after an interruption of data collection and case management in 2008 and 2009, an electronic alert system based on MEWS was implemented to identify patients at risk. During 2010, the alert was sent by e-mail to the hospital telephone service, which informed the nurses responsible for each ward. In-house computer technicians developed an algorithm to automatically calculate the MEWS score at each ordinary insertion of vital signs in the electronic health record. Due to technical limitations, the insertion of SODs was not mandatory in this phase. A score ≥ 3 constituted an alert in the wards and allowed for the early identification of at-risk patients.
- Direct electronic alert to nurse's mobile phone: in 2011 and 2012, the electronic alert was sent by e-mail to mobile devices available to each hospital nurse responsible for the ward where the patients were hospitalized.
- From 2013 to 2015, (1) all fields for vital signs and SOD became mandatory in the electronic medical record, and (2) a maximum time window of 1 hour was allowed for the insertion of vital signs measured every 6 hours.

After the alert (manual or electronic), if the patient was identified with suspected sepsis during the nurse's evaluation, the hospitalist was called to evaluate the case and to implement appropriate treatment as necessary. All changes proposed for the implementation of the early-sepsis-risk-detection protocol were mostly operational, and it was not necessary to hire staff or make structural changes.

### Data collection and treatment

Epidemiological data, Acute Physiology and Chronic Health Evaluation II (APACHE II) score, hospital sector of diagnosis, time required from triage-to-diagnosis (time between the activation by the warning system and the diagnosis by the hospitalist registered in medical records), time from triage-to-antibiotics (time between the activation by the warning system and the start of the antibiotic registered in medical records), intensive care unit (ICU) and hospital length of stay, and mortality were collected. Patients with end-stage diseases or shock due to noninfectious causes were excluded. The data collected were transferred to a spreadsheet for subsequent analysis. Inconsistencies and blank data were not considered in the analyses for categorical or continuous variables.

### Statistical analysis

All analyses were performed with MedCalc Statistical Software version 16.4.3 (MedCalc Software bvba, Ostend, Belgium). Categorical variables were expressed as the absolute and relative frequencies and compared using the chi-squared test. Continuous variables were expressed as the medians and interquartile ranges (IQR) and analyzed with a Kruskal-Wallis test and the Bonferroni-corrected pairwise Mann-Whitney test for post hoc analysis. P values < 0.05 were considered statistically significant.

The study protocol was approved by the Ethics Committee of *Hospital Municipal São José* of the Joinville (SC) under registration number CAAE 51661515.3.0000.5362.

## RESULTS

In total, 637 septic patients were detected over the course of the institutional sepsis program. Of all patients with sepsis, 309 (48.5%) were male. The median age was 62 (57 - 68) years, and the median APACHE II score was 20 (18 - 23). There were 9.4 daily electronic alerts on average over the years, ranging from 6.2 to 11.6. The ratio of the number of medical evaluations to the number of electronic alerts was 1:5; for every 4.1 patients evaluated by the physician, one was diagnosed with sepsis.

As shown in table 1, the distributions of age, sex and APACHE II score were similar over time. After the implementation of electronic screening in 2010, there was a significant increase in antibiotic administration in the first hour after the diagnosis of sepsis, collection of blood for hemocultures before antibiotic administration and serum lactate dosage in relation to the manual surveillance for clinical signs of infection (2007).

**Table 1:** Demographic, epidemiological and clinical data of septic patients

	2006 (N = 34)	2007 (N = 59)	2010 (N = 95)	2011 (N = 101)	2012 (N = 75)	2013 (N = 100)	2014 (N = 82)	2015 (N = 91)
Male gender	20 (58.8)	31 (52.5)	45 (47.3)	51 (50.4)	36 (48.0)	49 (49.0)	30 (36.5) ††	47 (52.2)
Age (years)	64 (58 - 73)	63 (60 - 68)	66 (61 - 69)	63 (57 - 69)	57 (51 - 63)	59 (54 - 64)	57 (53 - 62)	64 (60 - 69)
APACHE II	23 (21 - 26)	20 (18 - 22)	20 (18 - 23)	20 (16 - 21)	21 (18 - 24)	20 (18 - 23)	20 (18 - 22)	19 (18 - 24)
Source of infection								
Pulmonar	10 (29.4)	22 (37.3)	29 (30.5)	31 (30.6)	26 (34.6)	27 (27.0)	16 (19.5)††	28 (31.1)
Urinary	7 (20.6)	7 (11.8)	30 (31.5)	31 (30.6)	12 (16.0)**	30 (30.0)††**	18 (21.9)	26 (28.8)††
Abdominal	10 (29.4	20 (33.9)	20 (21.0)	17 (16.8)	12 (16.0)††	20 (20.0)	21 (25.6)	15 (16.6)††
Others	7 (20.5)	10 (17.0)	16 (16.8)	22 (21.7)	25 (33.3)††	23 (23.0)	27 (32.9)††	21 (25.5)
Septic shock	23 (67.7)	36 (61.0)	54 (56.8)	66 (65.3)	56 (74.6)	57 (57.0)**	43 (52.4)	37 (41.1)††
Antibiotic in the first hour	13 (38.2)	25 (42.3)	46 (48.4)	83 (82.1)†*	59 (78.6)†	71 (71.0)†	61 (74.3)†	66 (73.3)†
Blood culture prior to antibiotic	18 (52.9)	29 (49.1)	80 (84.2)†	76 (75.2)†	61 (81.3)†	85 (85.0)†	70 (85.3)†	83 (92.2)†
Lactate dosage	20 (58.8)	40 (67.7)	93 (97.8)†	96 (95.0)†	74 (98.6)†	92 (92.0)†	67 (81.7)††**	73 (81.1)
MAP ≥ 65mmHg in 6 hours	-	-	46 (78.4)	78 (77.2)	66 (88.0)	85 (85.0)	73 (89.0)	72 (80.0)

APACHE II - Acute Physiology and Chronic Health Evaluation II; MAP - mean arterial pressure.

* p < 0.001 and

** p < 0.05 for comparison in relation to the previous year.

† p < 0.001 and

†† p < 0.05 for comparison of variables after the implementation of electronic screening (2010) in relation to the manual active surveillance for clinical signs of infection (2007). The results expressed as n (%) or median (interquartile ranges).

Table 2 shows that, after implementation of the manual surveillance system for clinical signs of infection, the median triage-to-diagnosis time was reduced from 19:20 (9:10 - 38:15) hours to 12:40 (2:50 - 23:45) hours (p = 0.14). After the adoption of electronic alerts in 2010, an additional reduction to 2:10 (1:25 - 2:20) hours was observed when the alert was automatically sent to the hospital telephone service (p = 0.014) compared to manual surveillance (2007). In 2011, a greater decrease to 1:00 (0:30 - 1:10) hour was obtained when the alert was sent directly to the nurse's mobile phone on the wards (p = 0.016), remaining below this time interval over the following years (p < 0.01). The diagnosis-to-antibiotic reduced from 1:00 (0:55 - 1:30) hour in 2010 to 0:45 (0:30 - 1:00) minutes in 2011 (p = 0.02), maintaining similar values over the following years. The mean triage-to-antibiotic time was also reduced significantly from 2:25 (1:55 - 3:50) hours in 2010 to 1:50 (1:10 - 2:00) hours in 2011 (p < 0.001) and remained around this value in the following years.

**Table 2:** Elapsed times from triage-to-diagnosis, diagnosis-to-antibiotics and triage-to-antibiotics over the years

	2006 (N = 34)	2007 (N = 59)	2010 (N = 95)	2011 (N = 101)	2012 (N = 75)	2013 (N = 100)	2014 (N = 82)	2015 (N = 91)
	None	Manual surveillance	Indirect alert to the telephonist	Direct alert to nurse mobile phone		Direct alert to nurse mobile phone and mandatory insertion of vital signs and SOD in the EMR		
Triage-to-diagnosis time (hour)	19:20 (9:10 - 38:15)	12:40 (2:50 - 23:45)**	2:10 (1:25 - 2:20)**††	1:00 (0:30 - 1:10) **††	0:45 (0:30 - 1:00)†	0:30 (0:30 - 1:00)†	0:35 (0:15 - 0:50)†	0:35 (0:15 - 0:45)†
Diagnosis-to-antibiotic time (hour)	Unavailable	Unavailable	1:00 (0:55 - 1:30)	0:45 (0:30 - 1:00)**	0:45 (0:30 - 0:55)	0:30 (0:25 - 0:50)	0:30 (0:25 - 0:45)	0:40 (0:25 - 1:00)
Triage-to-antibiotic time (hour)	Unavailable	Unavailable	2:25 (1:55 - 3:50)	1:50 (1:10 - 2:00)*†	1:25 (0:55 - 1:30)**†	1:25 (1:10 - 1:40)†	0:50 (0:45 - 0:55)†	1:35 (1:15 - 1:40)†

SOD - signs of organ dysfunction; EMR - electronic medical record.

* p < 0.01 and

** p < 0.05 for comparison in relation to the previous year.

† p < 0.01 and

†† p < 0.05 for Kruskal-Wallis test for comparisons of variables after the implementation of electronic screening (2010) in relation to the manual active surveillance for clinical signs of infection (2007). The results expressed as the medians (interquartile ranges).

The sepsis-related in-hospital mortality showed a downward trend after implementation of the manual surveillance system (50.0% *versus* 32.2%, p = 0.09). In 2010, after the protocol interruption, hospital mortality increased again (37.9%), but not significantly. Compared to 2010, the reduction of hospital mortality did not reach statistical significance in 2011 (29.0%) or 2012 (26.6%). In the following years, further decreases were observed: 20.0% in 2013, 21.9% in 2014, and 24.1% in 2015 (p < 0.05). Intensive care unit and hospital length of stay were similar over time (Table 3).

**Table 3:** Outcomes of septic patients in the period

	2006 (N = 34)	2007 (N = 59)	2010 (N = 95)	2011 (N = 101)	2012 (N = 75)	2013 (N = 100)	2014 (N = 82)	2015 (N = 91)
ICU length of stay (days)	10 (4 - 13)	11 (8 - 16)	7.5 (4 - 11)	6 (5 - 10)	6 (4 - 8)	7 (5 - 9)	9 (5 - 12)	9 (6 - 15)
Hospital length of stay (days)	17 (11 - 32)	23 (16 - 33)	19 (15 - 26)	20 (14 - 27)	14 (11 - 17)	16 (12 - 20)	18 (12 - 25)	17 (13 - 22)
28-day mortality	5 (14.7)	10 (16.9)	16 (16.8)	14 (13.8)	17 (22.6)	14 (14.0)	11 (13.4)	17 (18.8)
In-hospital mortality	17 (50.0)	19 (32.2)	36 (37.9)	29 (29.0)	20 (26.6)	20 (20.0)†	18 (21.9)†	22 (24.1)†

ICU - intensive care unit.

† p < 0.05 for comparison of variables after the implementation of electronic screening (2010) in relation to the manual active surveillance for clinical signs of infection (2007). The results expressed as the medians (interquartile ranges) or n (%).

Comparing survivors and non-survivors by univariate analysis, the speed in screening patients at risk was similar (1:00 [0:45 - 1:10] *versus* 1:00 [0:45 - 1:25], p = 0.19) as were all other variables related to clinical management. The incidence of septic shock, as well as median age and APACHE II, was higher among non-survivors (Table 4).

**Table 4:** Comparison between survivors and non-survivors in the second phase

Variables	Survivors (N = 456)	Non-survivors (N = 181)	p value
Male	215 (47.2)	91 (50.3)	0.47
Age (years)	61 (58 - 62)	73 (69 - 76)	< 0.001
APACHE II (points)	18 (17 - 19)	26 (25 - 28)	< 0.001
Septic shock	154 (32.9)	110 (60.9)	< 0.001
Screening-diagnosis time (hour)	1:00 (0:45 - 1:10)	1.00 (0:45 - 1:25)	0.19
Antibiotic in the first hour	275 (60.3)	106 (58.6)	0.68
Blood cultures prior to antibiotic	370 (81.2)	155 (85.4)	0.19
MAP ≥ 65 mmHg in 6 hours	376 (82.5)	158 (87.2)	0.13
Lactate dosage	418 (91.7)	167 (92.3)	0.80
ICU length of stay (days)	6 (5 - 7)	6.5 (6 - 8)	0.11
Hospital length of stay (days)	14 (11 - 18)	16 (15 - 18)	0.71

APACHE II - Acute Physiology and Chronic Health Evaluation II; MAP - mean arterial pressure; ICU - intensive care unit. The results expressed as n (%) or median (95% confidence interval).

## DISCUSSION

Our institutional improvements using an early warning system for sepsis detection, including an electronic warning system, reduced the triage-to-diagnosis time and helped decrease and maintain lower diagnosis-to-antibiotic times over the study period. The adoption of electronic devices added efficiency to the previously adopted manual system for screening patients at risk. Targeting the alert to the telephone service reduced the time between triage and diagnosis. There was an even further reduction after adoption of mobile phones to deliver alerts to the nurses. In parallel, in the last three years, a reduction in mortality was observed.

In addition to facilitating patient data collection,^(41)^ some publications have suggested that electronic health records can help with the recognition of at-risk patients. Sawyer et al. observed earlier diagnostic and therapeutic interventions after septic patients were identified with an electronic alert system.^(34)^ Similarly, Kurczewski et al. demonstrated that the implementation of sepsis alerts reduced the time to any sepsis-related intervention.^(35)^ Umscheid et al. utilized an electronic health record that evaluated alterations of SIRS and organ dysfunction, as determined by lactate or hypotension. Patients received antibiotics earlier and more appropriately, more fluids were given, and blood cultures and lactate dosages were more frequently requested.^(42)^ Additionally, two randomized trials demonstrated that electronic warning systems are feasible and safe for at-risk patient detection in the ICU, but they may be more useful outside the ICU because the systems themselves were not sufficient to reduce changes in guideline compliance or mortality.^(36,37)^ Although no differences in clinical outcomes were demonstrated, all of these studies ensure safety to the care process.^(34-36,42)^

Irrespective of the screening method, manual or electronic, it is essential to establish triggers that alert specific situations to ensure the accuracy of the alerts.^(18)^ It is well known that the use of signs of inflammation alone to identify patients with sepsis has its limitations, mainly due to its high sensitivity and low specificity.^(23)^ On the other hand, considering its high specificity to detect patients at risk of death, qSOFA was found to be an overly specific tool for the detection of patients at risk of sepsis. With this perception, some authors have suggested adoption of the expanded clinical signs of infection, which bring together inflammatory signs and clinically detectable SOD to "track and trigger" critically ill patients with signs of deterioration.^(17,18,30,33)^ However, it is important to consider that "track and trigger" systems tend to achieve better outcomes when there is a sufficient mix of skills among the experienced staff. These electronic warning system protocols have to be used flexibly alongside clinical judgement, and staff need to have access to ongoing, multiprofessional and competency-based education.^(43)^

On average, for every 4.1 patients evaluated by the physician, at least one was diagnosed with sepsis. Our electronic warning system began with manual surveillance for screening patients with SIRS and/or SOD signs, and the latest version used the electronic alert based on MEWS score, an early warning system that combines SIRS and qSOFA signs.^(31,40,44)^ Early warning scores, such as MEWS, were more accurate than SIRS and qSOFA scores alone for identifying at-risk patients with a suspicion of infection from the ICU.^(45)^ Other studies have demonstrated high sensitivity and specificity in the detection of sepsis with the use of screening tools, both electronic and manual, but more studies are needed to determine the effectiveness of each type of alert.^(46-50)^ Despite this, there were no difficulties in the implementation or overload of the work of the medical or nursing staff.

It is difficult to determine if the electronic identification tool changed outcomes, considering that multiple changes in patient care occurred during the observation period. However, a role for early recognition in mortality reduction cannot be ruled out since the time to screening was the same in survivors and non-survivors. Both the time required from triage-to-diagnosis and triage-to-antibiotics as well as different aspects of early treatment of sepsis were similar between survivors and non-survivors. Considering that the efficiency of the care process offered to these two groups of patients was similar and that variables such as age, APACHE II and occurrence of septic shock were higher among non-survivors (Table 4), the intrinsic conditions of severely ill patients may carry more weight in the risk of death associated with sepsis and septic shock. The mortality rates higher than 30% observed in the first years are comparable to those found in Brazilian adult ICUs by the BASES Study and SPREAD Study.^(1,51)^

There was a significant increase in adherence to the 6-hour bundle observed over time, which was associated with a reduction in mortality that remained under 30% and can be explained by early diagnosis and appropriate treatment after implementation of the alert system. This rate is similar to those reported in a comparative meta-analysis, which showed a decline in rates from 35.9% in 1996 to 29.2% in 2009.^(52)^ In a previous publication, we reported our experience with a manual surveillance system that resulted in a strong reduction in the time required to detect sepsis risk and in mortality related to sepsis, although there was no difference in compliance with the 6-hour sepsis bundle.^(18)^ Similarly, Shiramizo et al. noted a decrease in mortality in septic patients from 41.4% to 16.2% despite a decline in compliance with the 6-h sepsis bundle.^(21)^ Another before-after study concluded that early sepsis recognition by ward nurses improved the odds of surviving over 30 days (odds ratio - OR 2.7, 95% confidence interval - 95%CI 1.6 - 4.6). Interestingly, the pre-intervention and post-intervention groups had the same probability of receiving appropriate antibiotics within 24 hours.^(30)^ Larosa et al. created a tool to identify non-ICU patients at risk of sepsis using a written screening form and an overhead alert system. The authors found better compliance with antibiotic use and lactate dosage in the group of patients screened with their form. In addition, the adjusted mortality rates were significantly lower in this group.^(53)^ Hence, early detection of sepsis leading to the early administration of antibiotics is the true reason for the difference in mortality observed in these studies.

Our study has several limitations. There was an interruption of data collection and case management in 2008 and 2009. Our data showed a trend of improvement in the diagnosis and treatment of sepsis between 2007 and 2010, which can be explained by the use of the electronic alert system and by the awareness of the attendance team in the hospital; there was no way to differentiate how much each one contributed to the improvement of outcome. During 2010, an electronic alert system based on MEWS was implemented to identify patients at risk; however, the insertion of SODs was not mandatory in this phase, which may have led to a reduction in the sensitivity of the method. Thus, a tendency to improve the diagnosis and treatment of sepsis from 2010 to 2012 may have been underestimated. Although we had many years of follow-up before and after implementation of the protocol, the observational nature of this study only allows us to infer associations between the time reduction and decrease in mortality rates. Furthermore, this program was performed in a single private center. A multicenter analysis of this type of tool in public hospitals is needed to confirm the benefits of electronic tools for the early detection of septic patients.

## CONCLUSION

The improvements in our warning system helped reduce the time necessary to perform the diagnosis of sepsis and the time to antibiotics. Migration to an electronic alert system played a key role in reducing the triage-to-diagnosis time. The reduction in mortality observed over time cannot be attributed to the warning system but rather is due to a series of improvements in the process of care for septic patients. With the reduction of sepsis diagnosis time in both survivors and non-survivors, intrinsic variables of the patients carry more weight in the risk of death associated with sepsis.
